# Endoplasmic Reticulum Stress in Chronic Obstructive Pulmonary Disease: Mechanisms and Future Perspectives

**DOI:** 10.3390/biom12111637

**Published:** 2022-11-04

**Authors:** Yue Yu, Ailin Yang, Ganggang Yu, Haoyan Wang

**Affiliations:** Department of Respiratory Medicine, Beijing Friendship Hospital, Capital Medical University, Beijing 100050, China

**Keywords:** endoplasmic reticulum stress, chronic obstructive pulmonary disease, inflammation, apoptosis, autophagy, therapeutic

## Abstract

The endoplasmic reticulum (ER) is an integral organelle for maintaining protein homeostasis. Multiple factors can disrupt protein folding in the lumen of the ER, triggering ER stress and activating the unfolded protein response (UPR), which interrelates with various damage mechanisms, such as inflammation, apoptosis, and autophagy. Numerous studies have linked ER stress and UPR to the progression of chronic obstructive pulmonary disease (COPD). This review focuses on the mechanisms of other cellular processes triggered by UPR and summarizes drug intervention strategies targeting the UPR pathway in COPD to explore new therapeutic approaches and preventive measures for COPD.

## 1. Introduction

Eukaryotic cells depend primarily on the endoplasmic reticulum (ER) for the biosynthesis, folding, and modification of membrane-bound and secreted proteins in eukaryotic cells. Generally, cells can maintain a dynamic equilibrium between protein biosynthesis and folding, a process known as ER homeostasis [1]. ER stress is generated by an abnormal accumulation of misfolded proteins in the ER, which occurs when various factors disrupt the ER homeostasis. These physiological and pathological factors include oxidative stress, nutrient deprivation, ischemia, hypoxia, glucose deprivation, viral infections, and a loss of calcium homeostasis. The cellular processes that defend against ER stress and cope with the accumulation of unfolded and misfolded proteins are collectively termed unfolded protein response (UPR) [2,3]. By boosting the folding capacity of ER proteins and enlarging the ER compartment size, UPR activation reverses the process of ER stress. Moreover, UPR activation promotes ER-associated protein degradation to eliminate misfolded proteins and restore ER homeostasis [4,5]. If these protective processes fail to restore proteostasis, prolonged or severe UPR activation leads to ER functional damage and initiates proapoptotic pathways leading to cell death [6,7].

A surge of evidence has emerged linking the pathology of various diseases to ER stress and UPR [8,9]. In recent years, an increasing number of studies have concentrated on the involvement of ER stress in the pathophysiology of chronic obstructive pulmonary disease (COPD). Numerous reports have denoted that ER stress is one of the main reasons for the early development of COPD and the leading cause of apoptosis of alveolar epithelial cells [10,11,12]. Pharmacological agents that reduce ER stress or inhibit specific signaling molecules in the UPR pathways have demonstrated some therapeutic advantages in animal models of COPD [6]. In this review, we highlight the link between the regulatory mechanisms of the three branches of UPR and COPD and summarize recent pharmacological intervention strategies targeting the UPR pathway in COPD. In addition, we discuss the potential links between UPR and the pathogenesis of, and treatment strategies for, COPD.

## 2. The Function of the ER

The ER is a continuous membrane system composed of tubules and sheets that extend from the nuclear membrane throughout the cell [13]. The ER is required for the synthesis, folding, and transport of secretory and membrane proteins, as well as lipid and steroid synthesis, carbohydrate metabolism, and calcium storage [14].

Approximately 30% of proteins are synthesized in the ER [15], and ER membrane-associated ribosomes translate and transfer proteins into the lumen of the ER and fold proteins into their unique three-dimensional structures [16]. The proteins destined for secretion must be appropriately folded and undergo various post-translational modifications with the aid of chaperones and folding enzymes. These modifications involve glycosylation, disulfide bond formation, and oligomerization [14]. Moreover, the ER has a significantly greater calcium content and a higher oxidizing redox potential than the cytosol [17,18], which facilitates proper folding, modification, and assembly of secreted proteins. However, even with several proteins and complexes specializing in adequate folding, some proteins still do not acquire the correct structural and functional forms and are misfolded. A portion of incompletely folded or misfolded proteins can be refolded by binding to calnexin after glycosylation modification, while unmodifiable unfolded proteins can be transported back to the cytoplasm through the ER-associated degradation (ERAD) pathway. Subsequent ubiquitination and degradation by the 26S proteasome ensure that abnormal peptides do not inadvertently enter the secretory pathway and affect cell function [19,20].

## 3. UPR Signaling

Protein folding in the ER can be influenced by a variety of stresses, and ER stress can occur when the amount of protein that has to be folded exceeds the ER’s ability to fold it [21]. To ensure proper protein folding and maintenance of the ER function, ER stress triggers a series of cellular signaling pathways, called the UPR, to reprogram gene transcription, mRNA translation, and protein modifications to eliminate unfolded or misfolded proteins and restore protein homeostasis [22,23].

**Figure 1:**
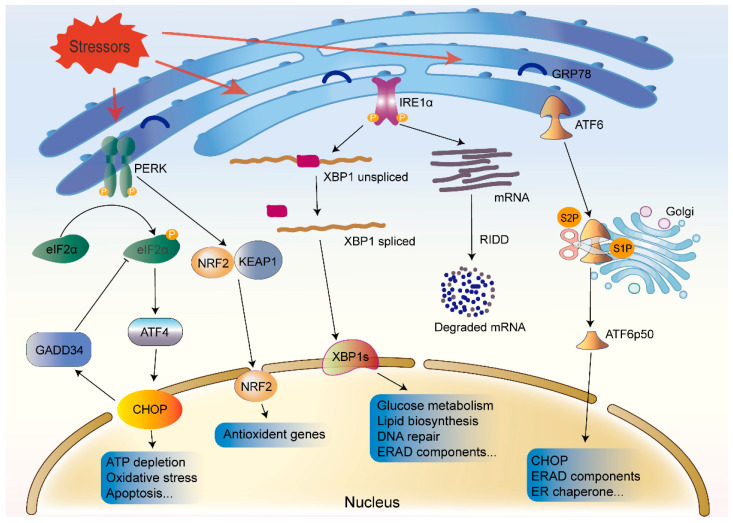
Endoplasmic reticulum (ER) stress-activated unfolded protein response (UPR) signaling pathways. When misfolded proteins accumulate in the ER, GRP78 dissociates from IRE1α, PERK, and ATF6 and then activates the downstream signaling cascades. **Left:** Activated PERK phosphorylates eIF2α, which upregulates the translation of ATF4. ATF4 can induce apoptosis by inducing CHOP. GADD34 transcription is upregulated by ATF4 and CHOP and leads to the dephosphorylation of eIF2α. PERK activation also induces the translocation of NRF2 to the nucleus to increase the transcription of antioxidant genes. **Middle:** IRE1α activation mediates unconventional splicing of the XBP1 mRNA. Spliced XBP1 (XBP1s) is involved in glucose metabolism, lipid biosynthesis, and DNA damage. IRE1α also degrades mRNA through regulated IRE1-dependent decay (RIDD). **Right:** ATF6 moves to the Golgi, where it is sequentially cleaved by S1P and S2P. The activated ATF6α (p50) fragment mediates the expression of CHOP and several components of ER-associated degradation (ERAD).

In mammalian cells, the UPR is primarily initiated by three ER transmembrane protein sensors, namely inositol-requiring enzyme 1 (IRE1) [24], protein kinase-like ER kinase (PERK) [25], and activating transcription factor 6 (ATF6) [26]. Under homeostatic conditions, the three ER stress sensors are combined with a chaperon, binding immunoglobulin protein (BiP; also known as glucose-regulated protein 78; GRP78) [27,28]. However, when misfolded proteins accumulate in the ER, it causes GRP78 to dissociate from IRE1, PERK, and ATF6 due to the high affinity of GRP78 for the exposed hydrophobic structural domains of the misfolded proteins [29,30]. The three sensors activate their downstream signaling cascades, slowing protein translation, eventually improving the folding capacity, and restoring ER homeostasis (Figure 1). Overall, the transcriptional program of the UPR acts as a complex signaling network that implements an adaptive program through multiple signaling cascades to restore ER homeostasis.

### 3.1. UPR Pathway Proteins

#### 3.1.1. IRE1

IRE1 is a type I transmembrane protein with two enzymatic activities, a serine/threonine kinase and an endoribonuclease, in its cytosolic domain [31]. In humans, IRE1 exists as two homologs, IRE1α and IRE1β [32]. In the UPR signaling pathway, IRE1α is a critical sensor involved in cell fate regulation. IRE1α is widely expressed, and IRE1α knockout mice exhibit embryonic lethality [33]; meanwhile, IRE1β is abundant only in intestinal and pulmonary epithelial and mucosal epithelial tissues, and IRE1β knockout mice survive [32,34]. GRP78 dissociation triggers IRE1 oligomerization in the ER membrane, facilitating trans-autophosphorylation of the IRE1 and activation of its cytoplasmic kinase domain [35,36]. Selective cleavage of X-box binding protein 1 (XBP1) mRNA by the activated IRE1 results in the expression of a functionally active spliced isoform (XBP1s) of the transcription factor XBP1 [15,37,38]. The XBP1s then translocates to the nucleus and drives the expression of genes involved in protein trafficking, folding, and the degradation of misfolded proteins to relieve ER stress and restore homeostasis [39]. XBP1s is also involved in glucose metabolism [40], lipid biosynthesis [41], redox metabolism [42], and insulin signaling [43]. It affects cell survival, differentiation, and development [35,44]. Moreover, XBP1s with transactivation domain modulates the expression of downstream target genes by combining with specific sites, such as hypoxia-response element reporter and G protein subunit alpha 12 [45,46].

In addition, IRE1 cleaves and downregulates mRNAs and microRNAs (miRNAs) with its RNase domain through a process known as regulated IRE1-dependent decay (RIDD) [47,48,49,50]. RIDD decreases the load of nascent proteins entering the ER and is essential for maintaining ER homeostasis and cell survival. Interestingly, the RIDD activity gradually increases during periods of heightened intensity or a long duration of ER stress. By contrast, ER stress-induced XBP1 mRNA splicing is independent of the intensity and duration of ER stress [51]. Under long-term persistent ER stress, XBP1 mRNA splicing is reduced, and RIDD activity is increased, aggravating uncontrolled cell death [48,51].

#### 3.1.2. PERK

PERK, a type I transmembrane protein kinase in the ER membrane, is activated through autophosphorylation and homodimerization after dissociating from GRP78 [25]. The activated PERK phosphorylates the downstream eukaryotic initiation factor 2α (eIF2α), which induces an overall downregulation of translation and inhibits protein synthesis [25]. Paradoxically, eIF2α phosphorylation elevates the translation of specific mRNAs with upstream open reading frames, such as transcription factor 4 (ATF4) [52]. ATF4 is a stress-induced transcription factor that induces apoptosis by initiating the transcription of C/EBP homologous protein (CHOP) under sustained stress conditions [53]. Furthermore, eIF2α phosphorylation is engaged in the translation of other ER stress-related proteins, such as those involved in growth stagnation. Growth arrest and DNA damage-inducible gene 34 (GADD34), which is positively regulated by phosphorylated eIF2α, is additionally transcriptionally induced by ATF4 and CHOP [54]. GADD34 is a modulatory subunit of the protein phosphatase 1C complex, which interacts to dephosphorylate eIF2α, thereby forming a negative feedback loop that restores protein synthesis [49]. Overall, PERK activation lessens the protein load in the ER, and, if the mechanisms involved are unable to restore ER homeostasis, PERK initiates cell death.

In addition, PERK phosphorylates nuclear factor erythroid 2-related factor 2 (NRF2) dissociates from Kelch-like ECH-associated protein 1 (KEAP1) and migrates into the nucleus. The translocation of NRF2 into the nucleus increases the production of antioxidant proteins, such as haem oxygenase 1 (HO-1), which aid in protein collapsing and restore ER homeostasis [32,55].

#### 3.1.3. ATF6

ATF6, a type II transmembrane protein, has two configurations, ATF6α and ATF6β, which show some functional redundancy [50,56]. Thus, ATF6α or ATF6β knockout mice can survive; however, embryonic lethality occurs when both the ATF6α and ATF6β are deleted [57]. ATF6α plays a dominant role in ER stress. Upon ER stress, GRP78 dissociates from the ATF6α, exposing the Golgi localization signal of ATF6α, which leads to the translocation of the ATF6α to the Golgi apparatus. In the Golgi, the full-length form of ATF6, ATF6α (p90), is sequentially cleaved by site-1 protease (S1P) and site-2 protease (S2P), liberating the cytoplasmic ATF6α (p50) segment [58]. The activated ATF6α (p50) fragment subsequently binds to cis-acting ER stress response elements in the nucleus, thereby promoting the expression of genes encoding proteins with functions to increase the ER capacity, including GRP78, GRP94, CHOP, and ERAD components, to restore protein folding homeostasis. Among these genes, CHOP depends on ATF6α rather than ATF4 alone for the transcription under ER stress. ATF6α can also induce increased expression of XBP1 and forms a heterodimer with XBP1 to reduce ER stress and increase the levels of genes required for ERAD [50,59].

### 3.2. The UPR and Other Cellular Processes

**Figure 2:**
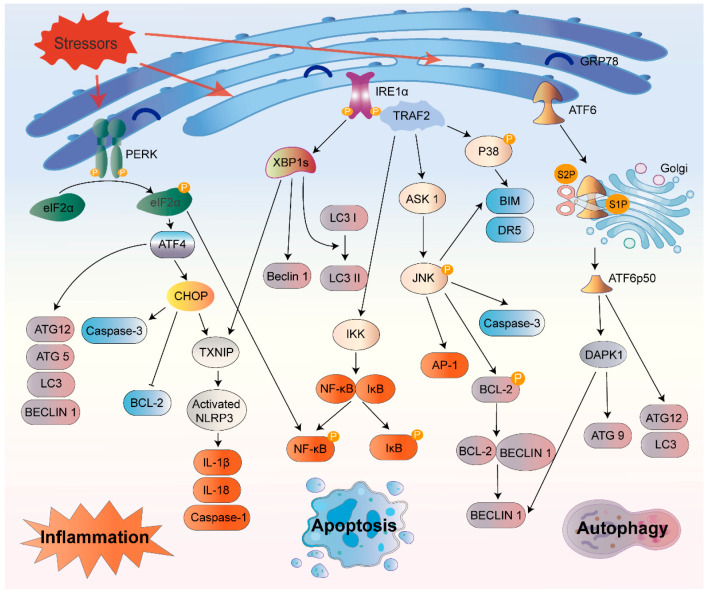
Mechanisms of unfolded protein response (UPR) signaling pathways triggered under endoplasmic reticulum (ER) stress. **Left:** Activated eIF2α can activate NF-κB by inhibiting the translation of IκB. ATF4 transcriptionally upregulates ATG12, ATG5, BECLIN-1, and LC3. CHOP can transcriptionally upregulate TXNIP1 and activate NLRP3 to increase the secretion of IL-1β and IL-18. **Middle:** IRE1α forms a complex with TRAF2, which activates ASK1 and IKK. Activated ASK1 phosphorylates JNK, leading to the subsequent activation of AP-1. Activated JNK promotes the expression of caspase-3, and BIM and can also phosphorylate BCL-2, leading to the release of BECLIN-1. Phosphorylation of IκB leads to the translocation of NF-κB to the nucleus, triggering inflammation. Cleaved XBP1 promotes the transcription of BECLIN-1 and the conversion of LC3 I to LC3 II and can also activate NLRP3 and increase the release of IL-1β and caspase-1. **Right:** Activated ATF6 transcriptionally upregulates ATG12 and LC3. Cleaved ATF6 induces the expression of DAPK1, which subsequently enhances ATG9 transcription and BECLIN-1 phosphorylation.

In response to prolonged ER stress, the UPR can start inflammatory responses, autophagy, ERAD, and apoptosis (Figure 2). However, if adaptive responses cannot restore protein folding homeostasis, UPR signaling eventually morphs into a terminal UPR that promotes cell death [18].

#### 3.2.1. Inflammation

Li and colleagues found that the onset of the UPR was adequate to prompt low degrees of inflammatory cytokine production, even without any obvious, irresistible stimuli [60]. Moreover, all three sensors of the UPR participate in the stimulation of inflammatory processes under ER stress. As a central transcriptional regulator, NF-κB is involved in multiple proinflammatory pathways. Normally, NF-κB forms a complex with the inhibitor ΙκΒ, which can translocate to the nucleus and initiate gene transcription. However, under ER stress, activated IRE1α binds with TNF receptor-associated factor 2 (TRAF2) to form a complex, which further activates apoptosis signal-regulating kinase 1 (ASK1) and IκB kinase (IKK). Subsequently, the IκB is phosphorylated and degraded, which eventually drives NF-κB translocation and triggers cytokine expression [61]. The IRE1α–TRAF2 complex can also activate c-Jun N-terminal kinase (JNK), which consequently phosphorylates and initiates the bZIP transcription factor activator protein 1 (AP-1) [62]. The activated PERK–eIF2α arm can restrain the translation of IκB and initiate the NF-κB pathway, prompting NF-κB nuclear translocation [63]. CHOP is activated within the PERK branch and enhances NF-κB signaling via transcriptional repression of the negative regulator peroxisome proliferator-activated receptor [64].

In addition to the induction of classical proinflammatory cytokines, there is mounting evidence that ER stress exacerbates the inflammasome-induced inflammatory cascade. The IRE1α/XBP1 pathway has been found to initiate NOD-, LRR-, and pyrin domain-containing protein 3 (NLRP3) inflammasome-mediated inflammation. In particular, XBP1 can activate the NLRP3 inflammasome, convert inactive caspase-1 to an active form, and promote IL-1β secretion to the extracellular space [65]. Active caspase-1 could trigger pyroptosis, a specific form of inflammatory cell death. An overexpression of CHOP activates the NLRP3 inflammasome and leads to pyroptosis [66]. The inhibition of ER stress could reduce the caspase-1 activity and rescue the cell from pyroptotic death [66]. Additional evidence indicates that the mechanism by which ER stress leads to inflammatory activation of NLRP3 is, at least in part, through thioredoxin-interacting protein (TXNIP), which is strongly induced downstream of IRE1α and/or PERK [23,67]. Interestingly, the UPR also interacts with the cytoplasmic peptidoglycan receptors NOD1 and NOD2 to induce the secretion of IL-6 [68].

#### 3.2.2. ERAD

Under ER stress, the UPR components activate two major protein degradation pathways: the ubiquitin–proteasome system (UPS) via ERAD and lysosome-mediated protein degradation via autophagy. ERAD is the process by which unfolded proteins are transported from the ER to the cytoplasm and degraded by the UPS [69]. Ubiquitination is essential for successfully targeting substrates for degradation. E3 ubiquitin ligase is a core component of the ERAD machinery, which connects multiple ER lumen and cytoplasmic junction proteins to translocate misfolded substrates into the cytoplasm for efficient degradation in the proteasome [20].

In mammalian cells, more than a dozen E3 ubiquitin–ligase enzymes are involved in ERAD. By ubiquitinating IRE1α, the E3 ligase, a mitochondrial ubiquitin ligase, can restrain ER stress-induced apoptosis [70]. Moreover, the E3 ligase carboxy-terminus of the HSC70-interacting protein (CHIP) induces IRE1α ubiquitination at Lys545 and Lys828 and optionally affects IRE1α phosphorylation and TRAF2 binding/JNK activation, thereby regulating ER stress-induced apoptosis and senescence [71]. In addition, the E3 ligase hydroxymethyglutaryl reductase degradation protein 1 (HRD1) is regulated by IRE1α and ATF6 [72]. A recent study has indicated that HRD1 could maintain T regulatory cell stability and function by inhibiting the IRE1α/p38-mediated ER stress response [73]. The phosphorylation of PERK and eIF2α is also crucial to the ERAD process. PERK activation can promote the phosphorylation of the E3 ligases MARCH5, MULAN, and Parkin, thereby increasing the degradation of related substrates [74]. HRD1 can promote eIF2α ubiquitination and proteasomal degradation to protect cells from apoptosis [75]. Furthermore, when ATF6α forms a heterodimer with XBP1s, the complex directs the expression of several ERAD machinery components [57,76]. ATF6 can regulate ERAD gene expression, which is essential for maintaining protein homeostasis [77].

#### 3.2.3. Autophagy

Autophagy is involved in many physiological processes and is essential for maintaining metabolic homeostasis. When misfolded or unfolded proteins accumulate beyond the ER capacity, autophagy can be induced as a secondary reaction to degrade the accumulated proteins, thereby alleviating ER stress. All three branches of the UPR can differentially regulate autophagy during ER stress. The IRE1α–TRAF2–ASK1 complex activates JNK, which leads to the phosphorylation of B-cell lymphoma-2 (BCL-2). This leads to the dissociation of BECLIN-1 from BCL-2, and the activation of the phosphatidylinositol 3-kinase (PI3K) complex promotes autophagy [78]. Moreover, JNK can directly regulate the expression of BECLIN-1 [79]. Notably, the autophagy-related protein (ATG) 12 can bind to and inhibit the BCL-2 protein, thereby promoting cell death [80]. Furthermore, the IRE1α/XBP1s axis increases the conversion of LC3 I to LC3 II in epithelial cells, leading to autophagy [81]. In addition, XBP1s triggers an autophagic signaling pathway through the transcriptional regulation of BECLIN-1 [82]. The PERK/eIF2α/ATF4 pathway appears to be the most critical pathway for inducing autophagy-related gene expression. It has been shown that PERK drives the expression of more than 10 autophagy genes, such as ATG12, ATG5, and BECLIN-1, to mediate autophagy through ATF4 [83]. Hypoxia-induced ER stress can lead to the upregulation of microtubule-associated protein-1 light chain-3β and ATG5 [84]. PERK/eIF2α/ATF4 signaling can also inhibit the mechanistic target of rapamycin (mTOR) complex I-activated autophagy by upregulating sestrin-2 [85]. Additionally, the depletion of ATF6 can decrease the transcription of ATG3 and BECLIN-1 [86] and inhibit autophagy via the mTOR pathway [87]. Another study has found that LC3 and ATG12 were transcriptionally upregulated through the ATF6 pathway [88]. ER stress can also trigger autophagy through ATF6/death-associated protein kinase (DAPK1)-mediated ATG9 transport and BECLIN-1 phosphorylation [89].

In summary, UPR and autophagy can be considered two programs for cellular homeostasis that work either independently or synergize to protect cells from various stresses. The induction of autophagy often acts as a protective mechanism. However, the overactivation of autophagy may be detrimental to cell survival. If misfolded proteins cannot be completely removed by autophagy and the UPS, they cause excessive ER stress, shifting the cell from survival to death mode.

#### 3.2.4. Cellular Senescence

Cellular senescence is an irreversible cell cycle arrest and increased secretion of inflammatory factors caused by various types of cellular stress, such as ER stress, mitochondrial dysfunction, and oxidative damage [90]. Studies have indicated that ER stress represses cyclin D1 and cyclin B1 expression through eIF2α phosphorylation and subsequent GADD45α induction, resulting in cell cycle arrest in G1/S and G2/M phases [91,92]. In addition, UPR-inducers induced senescence through the ATF6-cyclooxygenase 2 (COX2)/prostaglandin E2 (PGE2) axis; specifically, the silencing of the ATF6α and IRE1α decreased the expression of the COX2/PGE2 [93]. Dysfunctional autophagy has been reported to induce premature cellular senescence [94]. The evidence suggests that activation of ATF4 and CHOP, the molecular components of the ER stress pathway, can lead to cellular senescence through the induction of autophagy [95]. The inhibition of ER stress and autophagy could depress the expression of senescence markers and alleviate the senescence-associated secretory phenotype [90,96]. This evidence demonstrates that ER stress and autophagy in tandem play a crucial role in promoting cellular senescence.

#### 3.2.5. Apoptosis

When ER stress cannot be reversed, cellular functions deteriorate, frequently resulting in cell death. IRE1 is a critical molecule in the UPR signaling pathways. The activation of IRE1 also derepresses the translation of caspase-2 mRNA by degrading a specific miRNA, thus stimulating the mitochondrial apoptotic pathway [97]. In addition to inducing transcription of inflammatory factors, the IRE1α/TRAF2/ASK1 pathway causes the activation of downstream JNK and p38 mitogen-activated protein kinases (MAPKs), which promotes the apoptosis [98,99]. The ASK1−/− mice model displays reduced JNK activation and apoptosis under ER stress [100]. Activated JNK can promote the expression of apoptosis-related genes, such as caspase-3, and then initiate the death receptor or mitochondrial pathway to induce apoptosis [101]. BCL-2 and BCL2-like11 (BIM), respectively, are apoptosis-related substrates of JNK that are suppressed and activated by JNK phosphorylation [102,103]. In addition, p38 MAPK phosphorylation activates the transcription factor CHOP, which increases the expression of BIM and death receptor 5 (DR5) while decreasing that of BCL-2 to promote apoptosis [104]. Moreover, PERK overactivation can upregulate the CHOP/GADD153 transcription factor, which inhibits the antiapoptotic protein BCL-2 and enhances the expression of associated proapoptotic proteins [105]. A recent study has shown that the knockdown of CHOP and ATF4 by RNA interference can reduce the level of apoptosis in response to ER stress [106]. The transcription of CHOP is also regulated by ATF6 [107].

ER stress can also induce apoptosis through a caspase-dependent pathway. Caspase-12 is located in the outer membrane of the ER and is a critical molecule that mediates ER stress-related apoptosis. Similar to other caspases, caspase-12 exists in the form of an inactive zymogen. ER stress causes caspase-12 activation, and activated caspase-12 cleaves and activates caspase-9; in turn, activated caspase-9 activates caspase-3 and other caspases, ultimately leading to apoptosis [108]. A recent study has shown that ER stress-mediated apoptosis could be alleviated by inhibiting the IRE1α/XBP1s/caspase-12 pathway [109].

## 4. COPD and ER Stress

COPD is a progressive lung disease characterized by emphysema and chronic bronchitis. COPD is also associated with systemic inflammation, leading to multiple comorbidities and complications. Two major pathological processes that cause progressive airflow limitation in COPD are small airway remodeling and the destruction of the lung parenchyma [4,6]. The mechanisms of COPD are very complex and have not yet been fully elucidated. Increasing evidence indicates that ER stress is involved in the progression of airway inflammation and epithelial cell apoptosis in COPD, which are the essential mechanisms of early COPD pathogenesis.

Cigarette smoke (CS) is the most common risk factor for lung inflammation in COPD, which can predispose individuals to acute lung injury and pulmonary infections [110]. Some reactive intermediates, such as acrolein, are produced by the reactive oxygen species (ROS) and reactive nitrogen species present in CS. Their cytotoxicity causes oxidative damage and misfolding of proteins in the lung. Misfolded proteins that accumulate in the cells eventually cause ER stress [4].

### 4.1. CS-Induced UPR in COPD

Proteomic analysis showed that long-term smoking induced upregulation of several UPR chaperones and folding enzymes in human lung tissue [111]. Other studies have shown that GRP78 protein levels are increased in the lungs and serum of smokers and patients with COPD, as well as in the bronchoalveolar lavage fluid of smokers, which is connected to decreased lung function and emphysema severity [112,113]. Anti-GRP78 autoreactivity is a risk factor for atherosclerosis and osteoporosis in smokers [114,115]. Compared with those in long-term smokers, the lung levels of GRP78, calreticulin, and protein disulfide isomerase (PDI) are significantly lower in ex-smokers [111]. Weidner and colleagues [13] observed that the ER and endomembrane structures were disorganized in fibroblasts from patients with COPD, and these structural changes did not return to normal after several weeks of in vitro culture. This study suggests that quitting smoking can partially reverse protein unfolding, but structural changes in the lung lining of smokers are permanent, and this cellular fragility in response to stress may explain why some smokers develop COPD. In contrast to these studies, Korfei and colleagues [116] did not observe the expression of UPR-related signals in the lung tissues of patients with COPD, which indicates apparent differences in the expression of UPR-associated proteins in different individuals.

UPR activation was also detected in animal models of CS-induced COPD. Increased protein and mRNA expression levels of CHOP and GRP78 were detected in the lung tissues of COPD rats [117,118]. Long-term exposure to CS also increased the expression levels of the GRP78, ATF4, ATF6, and CHOP proteins and the phosphorylation of PERK and IRE1 in the lung tissues of mice [12]. With prolonged CS exposure, the expression of UPR-related proteins in the lung tissues of rats significantly increased [119]. Interestingly, Kenche and colleagues [120] confirmed that a single cigarette could lead to increased levels of phosphorylated eIF2α and ATF6 (p50) in the lung tissues of mice. Geraghty et al. [121] also demonstrated that acute exposure to CS caused an increase in CHOP levels in mice and guinea pigs. However, after long-term exposure to CS, CHOP expression in lung tissues decreased. In addition, CS exposure increased the expression of ER stress biomarkers in the diaphragms of rats. ER stress-induced apoptosis may be related to the atrophy of the diaphragmatic muscle caused by CS [122]. Unlike that in CS-exposed rats, no increased UPR signaling was found in the diaphragm muscles of stable patients with COPD [10]. This suggests that different individuals and species react differently to CS, and transcriptome analysis also revealed that few genes were universally regulated between mice and humans [123].

Several in vitro studies have demonstrated that exposure to CS extract (CSE) can trigger ER stress in the airway epithelial cells and other associated cells, such as lung cancer cells and alveolar macrophages [12,124,125]. Moreover, CSE exposure causes the oxidation of the ER chaperone PDI, which may contribute to the underlying mechanism of ER stress [126]. CS can also cause oxidative stress and severely damage proteins in the lungs. Exposure to gas-phase CS leads to decreased cystatin, trypsin, and tryptic rennin-like activities in human alveolar epithelial cells. The disruption of intracellular proteostasis under conditions of CS-induced ER stress is due to reduced proteasomal degradation of misfolded proteins, which results in the accumulation of damaged proteins and the reduction of the nascent protein synthesis [127]. Reduced proteasomal activity promotes CSE-induced aggresomal formation and increases apoptosis-inducing factor-mediated cell death [128].

### 4.2. CS-Induced UPR and Other Cellular Processes in COPD

#### 4.2.1. Inflammation

COPD may be caused by chronic exposure to smoke, dust, and other harmful particles, which induce chronic inflammatory responses in the airways, subsequently leading to irreversible pathological changes, such as small airway remodeling and pulmonary fibrosis. As previously described, all three UPR branches cause inflammatory responses. Significant ER stress was observed in the lung tissues of CS-induced COPD animal models, with elevations in the total inflammatory cell levels and neutrophil percentages in the bronchoalveolar lavage fluid and significantly increased levels of the inflammatory factors IL-6, IL-8, and TNF-α [12,118]. CS stimulation also activates the NLRP3 inflammasome, which promotes the secretion of IL-1β and IL-18 in the lung tissue [129]. Inhibitors or blockers of ER stress signaling can reduce CS-induced airway inflammation and improve emphysema [118,129,130].

#### 4.2.2. Cellular Senescence

Oxidative stress induced by excessive CS exposure can trigger lung cellular senescence through telomere shortening and DNA damage [131]. Acute/premature senescence plays an essential role in the pathogenesis of COPD by inducing changes in the systemic or local immune system and inflammatory factor secretion, interfering with tissue repair mechanisms after injury and weakening pulmonary defenses, among other complex mechanisms that impair lung function [132,133]. Fibroblasts and alveolar epithelial and endothelial cells from patients with COPD display shorter telomere lengths, DNA damage, accelerated cellular senescence, and oxidative stress [134,135]. The study has revealed that activation of UPR promotes the senescence of lung fibroblasts through the deletion of GADD34 [136]. However, studies on the interplay between ER stress and the UPR branch associated with cellular senescence in COPD still need to be further investigated.

#### 4.2.3. Apoptosis

CS exposure promotes the production of oxygen radicals, exacerbates changes in the intracellular redox status, and triggers oxidative stress. In vitro experiments have indicated that CSE stimulation elevates the expression of the apoptotic marker CHOP and induces caspase-dependent apoptosis [137,138]. Tagawa and colleagues [139] observed typical apoptotic manifestations, such as nuclear condensation, membrane blistering, and activation of the apoptosis-specific factors caspase-3 and caspase-4 in human bronchial epithelial cells after 24-h exposure to CSE. Subsequently, Tagawa et al. [140] found that CSE could trigger the PERK/eIF2α/CHOP pathway to induce apoptosis via superoxide anion. The inhibition of this pathway significantly suppressed the CSE-triggered CHOP expression and apoptosis.

The UPR also induces an elevated expression of antioxidants, especially NRF2. Under oxidative conditions, NRF2 translocates to the nucleus to combine with the antioxidant response element and induce the expression of endogenous antioxidant proteins and enzymes [141]. The NRF2 expression is elevated in the peripheral blood mononuclear cells in ex-smoking patients with mild to moderate COPD [142]. The upregulation of NRF2 alleviated CS-induced oxidative stress in rat lungs, attenuated CS-induced emphysema and airway remodeling, and reduced ER stress and apoptosis [143]. These results suggest that the upregulation of NRF2 expression may help prevent oxidative stress-related COPD progression. Interestingly, recent studies have shown that NRF2-deficient mice that were chronically exposed to particulate matter 2.5 had elevated oxidative stress and depressed ER stress levels but did not show obvious emphysema [144]. This may be attributed to the fact that the NRF2 deficiency reduces the expression of cytochrome P450 family 2 subfamily E member 1, thereby diminishing the cellular damage resulting from the metabolism of exogenous compounds [144,145].

#### 4.2.4. Autophagy

Most studies show that the autophagy mechanisms are impaired in COPD. Increased expression of p62 and LC3 in peripheral lung tissues from patients with severe COPD indicates impaired autophagy [146]. Likewise, alveolar macrophages from patients with COPD and smokers display large numbers of autophagosomes but impaired autophagic flux [147]. The same phenomenon was observed in CSE-stimulated human macrophage cell lines [148]. However, it has also been suggested that long-term exposure to CS causes enhanced autophagy in COPD mice, which is associated with COPD progression [149]. Autophagy is a complex regulatory process, and its increase or decrease during COPD progression may be related to different cell types, as well as differences in intensity and duration of stress.

Recent studies have revealed that the interaction between autophagy and ER stress plays an essential role in COPD progression. CSE stimulation induces apoptosis, autophagy, and ER stress-related protein expression in airway epithelial cells. The autophagy inhibitor 3-methyladenine was shown to increase the expression of CHOP, ATF4, and caspase-4, which correlated with CSE-induced apoptosis. Meanwhile, the ER stress inhibitor 4-phenylbutyric acid (4-PBA) inhibited autophagy. Furthermore, CSE-induced autophagy and apoptosis were enhanced by the knockdown of ATF4 or CHOP [150]. Hosaka and colleagues [151] further demonstrated the existence of functional crosstalk between UPR signaling and autophagy during CSE exposure. Impaired chaperone-mediated autophagy contributes to COPD pathogenesis by enhancing UPR-mediated epithelial apoptosis [151].

### 4.3. The Influence of Three Branches of UPR on the Pathogenesis of COPD

**Figure 3:**
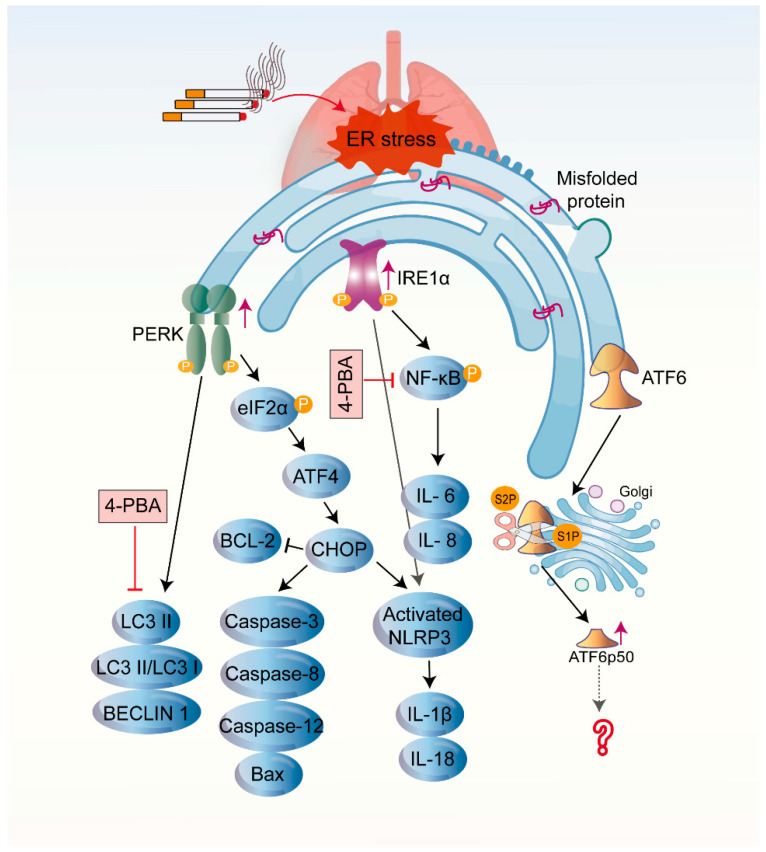
Cigarette smoke (CS)-induced unfolded protein response (UPR). Elevated expression of the three UPR sensors was detected in the lung tissue of smokers and animal models chronically exposed to CS. CS exposure could trigger activation of IRE1α, PERK, and their downstream signaling in airway epithelial and other associated cells, leading to airway inflammation and epithelial cell apoptosis, ultimately causing the development of COPD. The mechanisms of ATF6 involvement in COPD pathogenesis remain to be discussed. Endoplasmic reticulum stress inhibitor 4-PBA prevents the advancement of emphysema and airway inflammation by inhibiting autophagy and NF-κB signaling induced by prolonged CS stimulation.

Although studies have confirmed that activation of all three UPR branches can be observed in CS-induced COPD (Figure 3), the investigation of PERK and its downstream pathway signaling is the most extensive. CS-induced activation of the PERK/eIF2α/ATF4/CHOP pathway upregulated apoptosis factor caspase-3, caspase-8, caspase-12, and BCL-2 associated X (Bax) and downregulated the anti-apoptotic factor BCL-2 [137,143]. The action of various drugs is to reduce CS-induced apoptosis by inhibiting PERK, thereby alleviating emphysema. Moreover, CS-induced activation of the PERK pathway could trigger activation of the NLRP3 inflammasome, causing a series of inflammatory cascades that ultimately lead to a pulmonary inflammatory flare [129]. A knockdown of PERK also attenuated CSE-induced autophagy [150]. Efferocytosis is the process by which phagocytes remove apoptotic cells. Impaired efferocytosis is an essential mechanism in inflammatory lung disease. The evidence indicated that CS destroyed efferocytosis via the PERK/eIF2α pathway in macrophages [124].

In addition to modulating the NLRP3 inflammasome, IRE1α can promote CS-induced airway inflammation by activating the NF-κB signaling pathway [129,130]. Furthermore, the activation of IRE1α played a crucial role in the nicotine-induced epithelial-mesenchymal transition and decreased cell migration capacity in human bronchial epithelial cells [152]. Epithelial-mesenchymal transition is a pivotal contributor to airway remodeling in COPD. 

The ATF6 pathway is the least investigated in CS-induced COPD. CS-induced ATF6 cleavage was observed in lung lysates from mice [120]. However, some researchers have also found that the ATF6 pathway was inactivated in lung tissue from CS-induced COPD rats [137]. The studies related to ATF6 and its mediated signaling pathways in the pathogenesis of COPD are still insufficient. It is still remarkable that the endogenous cystic fibrosis transmembrane conductance regulator (CFTR) function is diminished under ER stress, which is associated with transcriptional repression of CFTR by ATF6 [153]. CFTR dysfunction can lead to significant impairment of mucociliary clearance and mucus hypersecretion, as well as to airway wall-thickening [154]. Mounting evidence demonstrates that acquired CFTR dysfunction is an essential contributor to the pathophysiology of COPD [154,155,156]. The reversal of CFTR dysfunction is an attractive therapeutic target for ameliorating the airway pathology in COPD [156]. Whether activation of the ATF6 pathway is correlated with CFTR dysfunction in COPD is unclear, and other mechanisms of ATF6 involvement in COPD disease progression need further investigation.

## 5. The UPR and Potential Therapeutic Interventions in COPD

The evidence of the COPD response to pharmacological manipulations of the UPR is limited to interventions in animal models and cells (Table 1). Wang and colleagues [130] demonstrated that 4-PBA prevented CS-induced emphysema, alveolar apoptosis, and inflammation by inhibiting ER stress and NF-κB signaling. Aggarwal and colleagues [157] indicated that the plasma haem levels and ER stress were elevated in patients with severe COPD and in a ferret model of COPD. Scavenging the haem with salubrinal (eIF2α dephosphorylation inhibitor) suppressed ER stress, reduced elastase levels and activity, and attenuated the development of fibrosis and emphysema pathophysiological phenotypes in COPD. Hydrogen sulfide (H2S), as an endogenous modulator, attenuated smoke-induced apoptosis and epithelial-mesenchymal transition by inhibiting ER stress, thus hindering the progression of lung function decline and emphysema formation in rats [152,158,159]. Melatonin counteracts CS-induced inflammasome activation and apoptosis by inhibiting ER stress and mitochondrial dysfunction [129,160]. Additionally, melatonin diminished CS-induced oxidative stress by upregulating the expression of the antioxidant NRF2 and improving the overall antioxidant status in the lungs of a COPD model [129]. Ursolic acid not only partially inhibits UPR signaling to alleviate ER stress-related apoptosis and oxidative stress but also abrogates CS-induced airway remodeling by modulating the three UPR pathways, which results in a therapeutic effect against COPD [137,143]. Epoxyeicosatrienoic acids (EETs) are produced via the cytochrome P450 epoxy synthase pathway and possess anti-inflammatory and antiapoptotic effects. Exogenous administration of 14,15-EET can prevent CSE-induced apoptosis by blocking UPR signaling [161]. Furthermore, adiponectin, a protein mainly secreted by adipocytes, alleviated the apoptosis of alveolar epithelial cells in COPD rats by suppressing ER stress [162]. Other reports have suggested that herbal ingredients, such as Fengbaisan, ephedrine, and curcumin, could modify COPD in vitro and in vivo by modulating ER stress [12,117,118,138,163].

Additionally, several vital mediators that regulate various processes may be involved in COPD by regulating ER stress. Sirtuin 1 (SIRT1) has become a focus of ER stress research in recent years. SIRT1 is mainly located in the nucleus and can enhance cellular stress resistance and boost cell survival in several ways. The upregulation of SIRT1 could inhibit ER stress and delay COPD progression [117,118,160,164]. AMP-activated protein kinase (AMPK) is an essential sensor of cellular energy status and has protective roles in various diseases. Liu et al. [165] showed that AMPK could activate oxygen-regulated protein 150 via the forkhead box O1 pathway, thereby blocking ER stress and preserving airway epithelial cells from CSE-induced apoptosis. Progranulin (PGRN) is extensively expressed in diverse epithelial cells and can restore epithelial tissue homeostasis in response to tissue injury. In a COPD model, PGRN suppressed apoptosis of airway epithelial cells by regulating the ER stress response and MAPK activation [166]. Furthermore, Chen et al. [167] confirmed that the silencing of orosomucoid 1-like protein 3 (ORMDL3), a transmembrane protein localized in the ER, abrogated smoking-induced human airway smooth muscle cell injury by repressing the UPR pathway. Moreover, miRNAs have been reported to be associated with the progression of many diseases, including COPD. A recent study indicated that miR-150-5p expression is decreased in patients with COPD and in COPD models. The overexpression of miR-150-5p could directly diminish CS-induced inflammation, facilitate cell migration, and achieve protective effects against COPD by targeting IRE1α [168].

## 6. Conclusions

In this review, we focus on the mechanisms of cellular processes triggered by CS-induced activation of UPR in the development of COPD. Each UPR sensor has a dual role, with some pathways of the initial UPR being adaptive and protective. When this early signaling fails to successfully restore ER homeostasis, the UPR may change into a signal that promotes cell death. UPR-related signals are elevated in patients with COPD and animal models, and a prolonged UPR may induce apoptosis of structural lung cells, ultimately leading to the development of emphysema and pulmonary fibrosis. However, ER stress signaling pathways are complex and tissue-specific, and the effects of ER stress on COPD depend on the different cell types and the degree of stimuli. Currently, pharmacological treatments targeting UPR signaling molecules exhibit great potential in delaying COPD progression. Based on recent research advances, we also found that targeted UPR exhibited remarkable therapeutic effects in COPD. Consequently, therapeutic strategies targeting ER stress may provide new directions for the treatment of COPD.

## Figures and Tables

**Table 1:** Therapeutic interventions for and key mediators of endoplasmic reticulum (ER) stress and the unfolded protein response.

	Experimental Model(s)	Reference(s)
ER stress inhibitor		
4-PBA	In vivo (murine); in vitro (BEAS-2B)	[130]
Salubrinal	Human subjects; in vivo (murine); in vitro (HBECs)	[157]
H_2_S	Human subjects; in vivo (rat); in vitro (16HBE)	[152,158,159]
Melatonin	In vivo (murine, rat); in vitro (L-132)	[129,160]
Ursolic acid	In vivo (rat)	[137,143]
14,15-EET	In vitro (BEAS-2B)	[161]
Adiponectin	In vivo (rat)	[162]
Herbal ingredients or mixtures	In vivo (murine, rat); in vitro (BEAS-2B, 16HBE, A549, HBECs, HFL1)	[12,117,118,138,163]
Key mediator		
SIRT1	In vivo (rat); in vitro (A549)	[117,118,160,164]
AMPK	In vitro (HBEpC)	[165]
PGRN	In vivo (murine); in vitro (A549)	[166]
ORMDL3	In vitro (HASMC)	[167]
miR-150-5p	Human subjects; in vivo (murine); in vitro (HBECs)	[168]

14,15-EET, 14,15-epoxyeicosatrienoic acid; 16HBE, human bronchial epithelial cell line; 4-PBA, 4-phenylbutyric acid; A549, human non-small cell lung cancer cell line; AMPK, AMP-activated protein kinase; BEAS-2B, bronchial epithelial cell line; HASMC, human aortic smooth muscle cells; HBECs, human bronchial epithelial cells; HBEpC, primary human bronchial epithelial cells; HFL1, human lung fibroblast cell line; L-132, the human lung alveolar epithelium cell line; ORMDL3, orosomucoid 1-like protein 3; PGRN, progranulin; SIRT1, sirtuin 1.

## Data Availability

Not applicable.
